# Action of antimicrobial photodynamic therapy with red leds in microorganisms related to halitose: Controlled and randomized clinical trial

**DOI:** 10.1097/MD.0000000000013939

**Published:** 2019-01-04

**Authors:** Ana Carolina Costa da Mota Ciarcia, Marcela Leticia Leal Gonçalves, Anna Carolina Ratto Tempestine Horliana, Ellen Sayuri Ando Suguimoto, Lysianne Araujo, Andreia Laselva, Marcia Pinto Alves Mayer, Lara Jansinsk Motta, Alessandro Melo Deana, Raquel Agnelli Mesquita-Ferrari, Kristianne Porta Santos Fernandes, Sandra Kalil Bussadori

**Affiliations:** a Doctoral Student of the Postgraduate Program in Biophotonics Applied to Health Sciences, Nove de Julho University; b Professor of the Postgraduate Program in Rehabilitation Sciences and of the Postgraduate Program in Biophotonics Applied to Health Sciences, Nove de Julho University; c Student of Scientific Initiation of Program in Biophotonics Applied to Health Sciences; d Microbiology ICB, University of São Paulo; e Professor of Microbiology ICB, University of São Paulo; f Professor of the Postgraduate Program in Biophotonics Applied to Health Sciences, Nove de Julho University; g Professor of the Postgraduate Program on Biophotonics Applied to Health Sciences of the Postgraduate Program in Biophotonics Applied to Health Sciences, Nove de Julho University, São Paulo, SP, Brazil.

**Keywords:** antimicrobial photodynamic therapy, halitosis, qPCR

## Abstract

**Introduction:**

Halitosis is the term used to describe any unpleasant odor relative to expired air regardless of its source. The prevalence of halitosis in the population is approximately 30%, of which 80 to 90% of the cases originate in the oral cavity resulting from proteolytic degradation by gram negative anaerobic bacteria. Antimicrobial photodynamic therapy (aPDT) has been widely used with very satisfactory results in the health sciences. It involves the use of a non-toxic dye, called photosensitizer (FS), and a light source of a specific wavelength in the presence of the environmental oxygen. This interaction is capable of creating toxic species that generate cell death. The objective of this controlled clinical study is to verify the effect of aPDT in the treatment of halitosis by evaluating the formation of volatile sulphur compounds with gas chromatography and microbiological analysis before and after treatment.

**Materials and Methods:**

Young adults in the age group between 18 and 25 years with diagnosis of halitosis will be included in this research. The selected subjects will be divided into 3 groups: G1: aPDT; G2: scraper, and G3: aPDT and scraper. All subjects will be submitted to microbiological analysis and evaluation with Oral ChromaTM before, immediately after treatment, 7, 14, and 30 days after treatment. For the evaluation of the association of the categorical variables the Chi-square test and Fisher's Exact Test will be used. To compare the means the student *t* test and analysis of variance (ANOVA) will be used and to analyse the correlation between the continuous variables the correlation test by Pearson will be applied. In the analyses of the experimental differences in each group the Wilcoxon test will be used. For all analyses a level of significance of 95% (*P* < .05) will be considered.

**Discussion:**

Halitosis treatment is a topic that still needs attention. The results of this trial could support decision-making by clinicians regarding aPDT using aPDT for treating halitosis.

## 1 Introduction

Halitosis is the term used to describe any unpleasant odor related to expired air regardless of its origin.^[1]^ The chemical components related to halitosis are volatile sulfur compounds (VSCs) such as hydrogen sulfide (H2S), methylmercaptanes (CH3SH), and dimethyl sulphide (CH3SCH3).^[2–5]^ The prevalence of halitosis in the population is approximately 30%, of which 80 to 90% of the cases originate in the oral cavity resulting from proteolytic degradation by gram negative anaerobic bacteria from sulfur-containing substrates in saliva, epithelial cells, blood, and food debris.^[6]^

The dorsum of the tongue and periodontal pockets are related as the major niches of bacteria that are responsible for the emission of VSCs. Some methods are used to diagnose halitosis: the organoleptic method, which is a subjective evaluation of the exhaled air of the mouth and nose, and a scale, is used to quantify this odor, depending greatly on the olfactory capacity of the evaluator. Sulphide monitors are devices that quantify the total value of VSC exhaled from the oral cavity. Gas chromatography is the most appropriate method to detect halitosis of different origins because it performs individual measurement of the three main gases (sulfhydride, methylmercaptanes, and dimethyl sulphide), allowing to assess the intensity of the breath and its origin. However, the lack of standardization in the protocol for diagnosis and treatment of halitosis makes it difficult to compare the epidemiological data obtained in different countries.^[7,8]^

Currently the treatment of halitosis is related to the masking of the odor or the decrease of the number of bacteria, through chemical and mechanical removal with the use of tongue scrapers and mouthwashes.^[5,9–13]^ Antimicrobial photodynamic therapy (aPDT) has been widely used with very satisfactory results in the health sciences. It involves the use of a non-toxic dye, called photosensitizer (FS), and a light source of a specific wavelength in the presence of the environmental oxygen. This interaction can create reactive oxygen species (ROC) that generate cell death.^[14–17]^ The advantages of this approach are to avoid resistance to target bacteria and damage to adjacent tissues as the antimicrobial effect is confined only to the areas covered by the photosensitizer and irradiated by light acting on the target organism rapidly, depending on the dose of light energy and power output. In addition, according to Wainwright, M.^[18]^ bacterial resistance to aPDT is unlikely, since the singlet oxygen and free radicals formed interact with various bacterial cell structures and different metabolic pathways.

Halitosis is considered one of the problems related to the impact of oral health on quality of life and is enough to affect the individual's perception about his life.^[19]^ Therefore, there is a need for new treatment alternatives for the patient, especially in the face of the technological advances in dentistry, so the patient's expectation regarding the final results can be met. The present study proposes to conduct a controlled clinical trial to evaluate the effectiveness of the application of aPDT in the tongue coating as a new way to control halitosis, mainly because it is a fast, non-invasive procedure and does not generate bacterial resistance.

## 2 Materials and methods

The study will follow the regulatory norms of research in humans with favourable opinion of the Research Ethics Committee of the University Nove de Julho number, and the participants will sign the informed consent form after clarifications for authorization of the participation in the research, according to Resolution 466/12 of the National Health Council. Type of study: controlled, quantitative, cross-sectional clinical study.

### 2.1 Hypothesis

Null hypothesis: There is no alteration of halitosis after the use of photodynamic therapy employing the use of blue dye and red LED. There is no microbiological alteration after antimicrobial photodynamic therapy. Experimental hypothesis: there is a decrease in halitosis after the use of photodynamic therapy using blue dye and red LED associated or not with the tongue scraper. There is microbiological alteration after antimicrobial photodynamic therapy.

### 2.2 Research subjects

For this study, young adults of both sexes, students and employees of the *Universidade Nove de Julho*, São Paulo will be evaluated.

### 2.3 Inclusion criteria

Young adults between the ages of 18 and 25, with an informed consent form and authorization for the diagnosis and treatment of halitosis. Young adults diagnosed with halitosis presenting Oralchroma S2H ≥ 112 ppb and/or CH3SH ≥ 26.

### 2.4 Exclusion criteria

Individuals will be excluded from the study:

- With dentofacial anomalies, in orthodontic and/or orthopedic treatment,
- Using a removable device, implant and/or prosthesis,
- With periodontal disease,
- With carious lesions,
- In cancer treatment,
- On antibiotic treatment up to 1 month before the survey,
- Pregnant,
- With hypersensitivity to the photosensitizer to be used.

**Figure 1:**
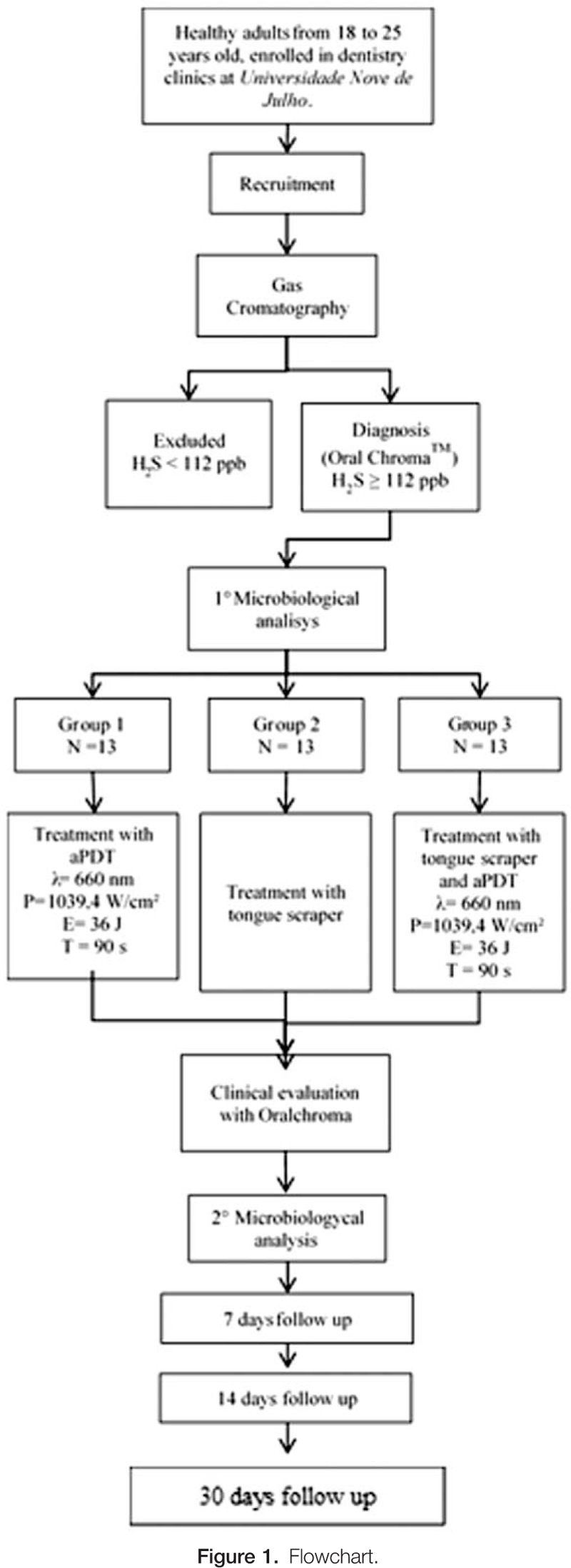
Flowchart.

As the research is a randomized clinical study and seeking greater transparency and quality of this research, we will use the CONSORT (Consolidated Standards of Reporting Trials) recommendations (Fig. 1). The protocol is in accordance with the 2013 Standard Protocol Items: Recommendations for Interventional Trials (SPIRIT) Statement.

The selected subjects will be divides by block randomization into 3 groups (n per group = 13), according to the treatment to be performed. Opaque envelopes will be identified, and a sheet containing the information of the corresponding experimental group will be inserted. There will be a 1 session for each group (Table 1). All samples from the subjects will be submitted to microbiological analysis and evaluation with Oral ChromaTM before and after treatment, followed by controls of 7, 14, and 30 days.

**Table 1:**
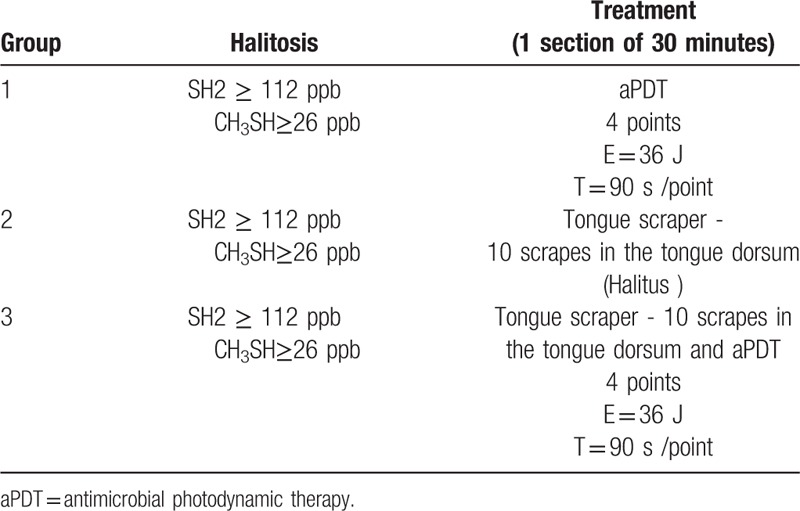
Experimental condition.

### 2.5 Microbiological analysis

Samples of tongue coating will be collected using a sterile swab that will be passed on the surface of the tongue dorsum with a single movement and light pressure. Samples will be deposited in sterile tubes that will be identified and stored at -80°C until analysed. After thawing, the samples will be vortexed for 1 minute. For extraction of the bacterial DNA samples will be boiled for 10 minutes and then centrifuged at 10,000 rpm for 10 minutes. The supernatant will be placed in a new microtube containing 100 μL phenol/chloroform/isoamyl alcohol (25: 24: 1), followed by ethanol precipitation. The purified DNA will be resuspended in TE buffers. The levels of P. gingivalis, T. forsythia, and T. denticola will be analysed by quantitative PCR. The quantitative analysis will be performed using real-time PCR using the Step One Plus Real-Time PCR System (Applied Biosystem, Foster City, CA) and fluorescently detected products using the Quantimix Easy SYG Kit (Biotools, Madrid, Spain), following the protocol recommended by the manufacturer. To the reaction 10l will be used SYBR Green 0.5 ul DNA template, 200 mM of each primer (P. gingivalis CATAGATATCACGAGGAACTCCGA TT and AAACTGTTAGCAACTACCGATGTGG; T. forsythia GGGTGAGTAACGCGTATGTAACCT and ACCCATCCGCAACCAATAAA, T. denticola CGTTCCTGGGCCTTGTACA and TAGCGACTTCAGGTACCCTCG; Universal bacteria CCATGAAGTCGGAATCGCTAG and GCTTGACGGGCGGTGT) in total volume of 20 μl. For the standard curve, reactions containing template DNA 2 to 2X105 copies of the analyzed gene (16S rRNA) will be performed using pTOPO plasmids in which the 16S genes of the 14 different organisms will be cloned. As a negative control sterile milliQ water will be added instead of DNA template. Reactions to 16S rRNA will be performed with initial denaturation at 95°C for 2 minutes followed by 36 cycles of 94°C for 30 seconds, 55°C for 1 minute and 72°C for 2 minutes and final extension at 72°C for 10 minutes 46. Fluorescence will be detected after each cycle and plotted using Step One Plus Real-Time PCR System software (Applied Biosystem, Foster City, CA). To ensure the specificity of the products detected by fluorescence and to avoid detection of primer dimers, the detection will be performed to a degree below the dissociation temperature of the amplicons. All samples will be analyzed in duplicate and each dilution of the plasmids to the standard curve in triplicate. The purpose of the microbiological evaluation will be to verify the effectiveness of the photodynamic therapy for the treatment of halitosis, complementing the clinical evaluation.^[20]^

### 2.6 Assessment of halitosis level

**Figure 2:**
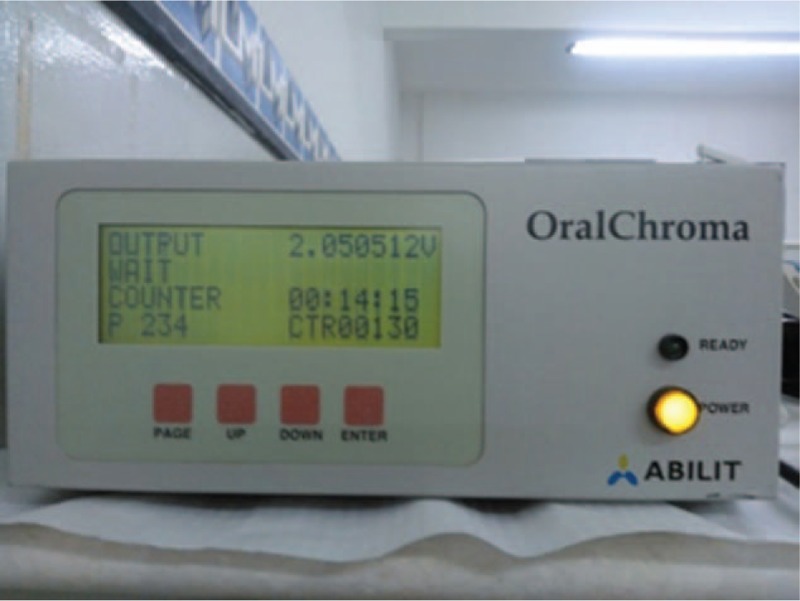
Oral Chroma.

The literature describes some methods for measuring halitosis, such as the organoleptic assessment of air emanated from the oral cavity,^[21,22]^ by sulfide monitor^[21,23,24]^ and by gas chromatography.^[24–26]^ However, it has already been demonstrated that the organoleptic test can be influenced by the olfactory ability, the emotional state of the examiner and by climatic conditions. Therefore, the Oral Chroma portable device (Abilit, Japan) (Fig. 2), using a highly sensitive semiconductor gas sensor, will be used for this study.

**Figure 3:**
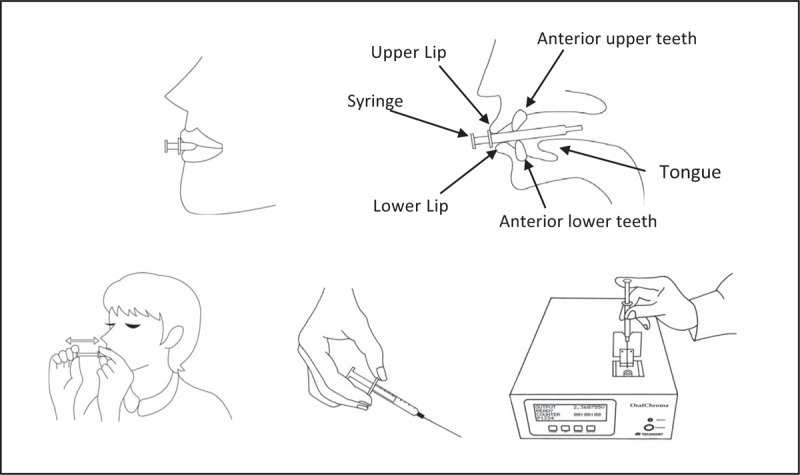
Halimetry procedure.

The air collection will follow the OralChromaTM Manual Instruction, in which the participant is instructed to make a mouthwash with cysteine (10 mM) for 1 minute to differentiate the VSCs origin and remain mouth closed for 1 minute. A manufacturer's own syringe for collection of mouth air will be introduced into the patient's mouth with the plunger fully inserted. The patient closes his mouth, breathes through his nose and waits with his mouth closed for 1 minute. They will be asked not to touch the tip of the syringe with their tongue. The plunger will be pulled out, re-emptied into the patient's mouth and pulled out again to fill the syringe with the breath sample. The tip of the syringe will be cleaned to remove moisture from the saliva, the gas injection needle will be placed in the syringe and the plunger will be adjusted to 0.5 ml. The air is injected into the door of the appliance in a single movement (Fig. 3).

OralChromaTM allows the capture of gas concentration values by measuring the VSC thresholds (from 0 to 1000 ppb), very accurately after 8 minutes. The results are stored on the device itself and can be retrieved and viewed at any time for comparison before and after treatment.

From the analysis of VSC captured by the system, we have:

- Sulfide: originates mainly from the bacteria present on the back of the tongue. Values above 112 ppb are indicators of halitosis.
- Methylmercaptanes: predominantly higher in the periodontal pockets. Values up to 26 ppb are considered normal. Periodontal disease typically results in a high ratio of methylmercaptanes/sulfhydride (> 3:1)
- Dimethyl sulphide: may have periodontal or systemic (intestinal, hepatic, pulmonary) origin. It can also be caused, temporarily, by the ingestion of certain foods and beverages. The perception threshold of dimethylsulphide is the lowest, 8 ppb.

To avoid changes in halimetry, the examination will be conducted in the morning^[26]^ and participants will be instructed to follow the following guidelines: 48 hours before the evaluation, avoid ingesting food with garlic, onion and strong seasoning, alcohol consumption and use of oral antiseptic. On the day of the evaluation, in the morning, you can eat up to 2 hours before the examination, abstain from coffee, candies, chewing gum, oral hygiene products, and perfumed personnel (aftershave, deodorant, perfume, creams and/or tonic) and the tooth brushing should be done only with water.^[27]^

### 2.7 Application of aPDT

**Figure 4:**
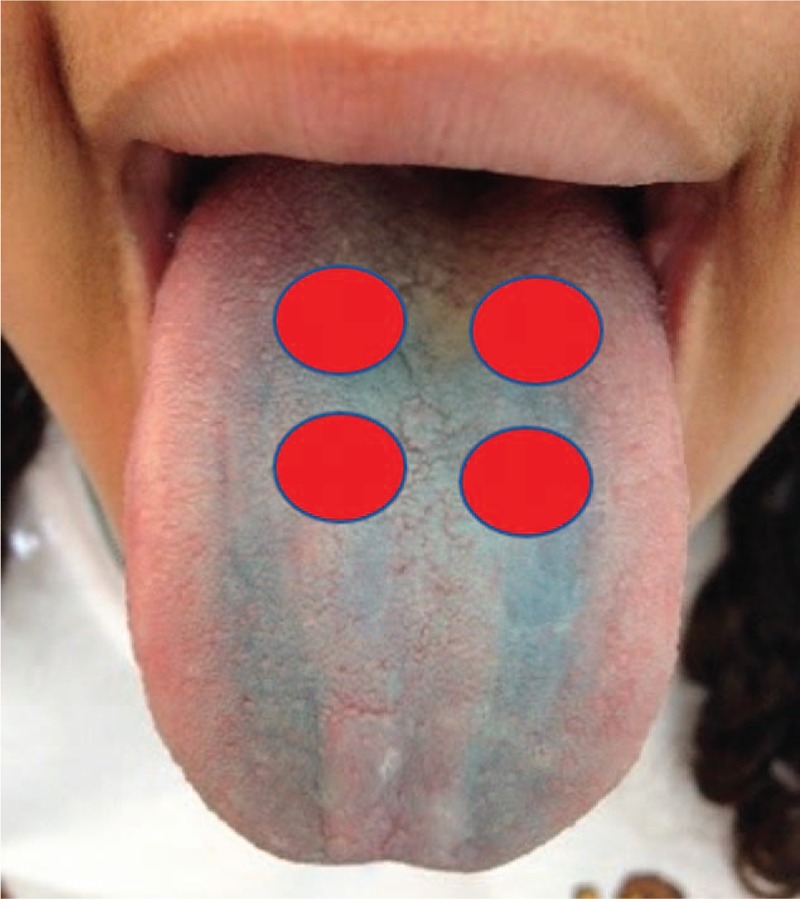
Implementation points for the aPDT. aPDT = antimicrobial photodynamic therapy.

**Figure 5:**
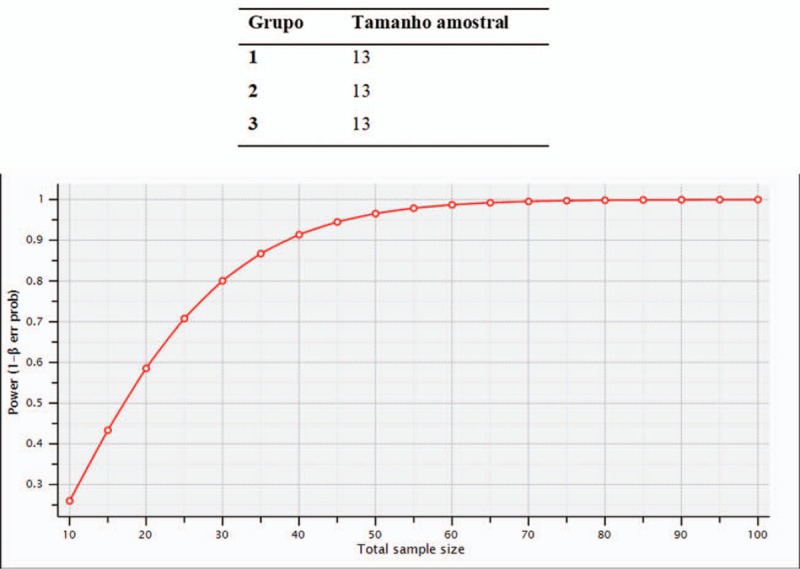
Shows an adjustment of the power of the test as a function of the total sample size.

For the photodynamic therapy, an equipment developed for this project will be used, with emission of red LED (660 nm) and tip of 2.84 cm^2^ in diameter. At the moment of application of the aPDT, only the volunteer to be treated and the professional responsible will be present, both using specific eye protection glasses. The active tip of the laser will be coated with clear disposable plastic (PVC) (avoiding cross contamination) and the professional will be properly dressed (Fig. 4). Methylene blue will be used as the photosensitizing agent, at a concentration of 0.005% (165 μm), to be applied in enough quantity to cover the middle third and back of the tongue for 2 minutes for incubation. The excess will be removed with a sucker in order to maintain the surface wet with the photosensitizer itself, without the use of water. Four points will be irradiated with 1 cm between them, considering the scattering halo and the effectiveness of aPDT (Fig. 5). Based on previous studies carried out with aPDT for the treatment of halitosis^[21–24]^ the apparatus will be previously calibrated with wavelength 660 nm, with energy of 36 J, power of 400 mW for Groups 1 and 3 that will be irradiated for 90 seconds per point, creep of 1039.4 mW/cm^2^ (Table 2).^[28]^

**Table 2:**
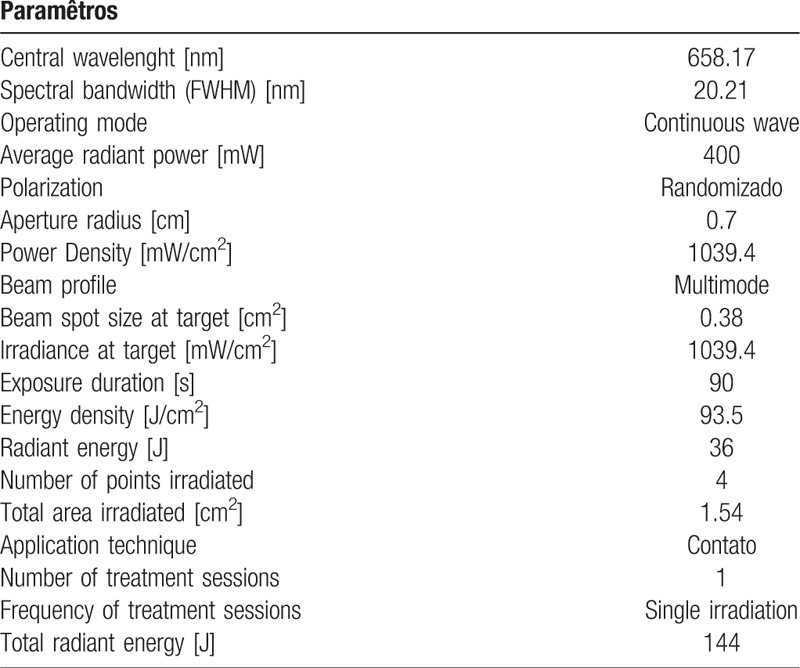
Shows the radiometric and spectroscopic parameters of the equipment developed for this project.

The method of point application will be used in direct contact with the tongue.

### 2.8 Sample calculation

For the calculation of the sample size, the data of the work by A. Costa Costa and Mota et al was used. Effect of photodynamic therapy for the treatment of halitosis in adolescents—a controlled, microbiological, clinical trial.^[29]^

Initially we established an error 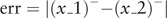, where 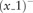 and 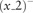 are the baseline values for periodontal treatment with PDT. From this error, the effect size, given by

err/√ (σ_1 ^ 2 + σ_2 ^ 2)

where σ_1 ^ 2 and σ_2 ^ 2 are the variances of groups 1 and 2, respectively.

Assuming that the studied groups have a normal or approximately normal distribution, that the sample size will be sufficiently large and that a 2-tailed test will be used, for a significance level α = 0.05 and maintaining the power of the test 1-β = 0.90 we have: In Figure 5 it is observed that with a total sample size of 39 subjects, that is, 3 groups with 13 samples each, the statistical difference should be demonstrated, keeping the power of the test greater than or equal to 0.90. If the normal distribution hypothesis is rejected, the sample size should be corrected by approximately 5%.

### 2.9 Organization and statistical processing of data

The data will be tabulated and treated in the program Bioestat 5.0. The descriptive statistics of the data will be performed. For the evaluation of the association of categorical variables, the Chi-square test and Fisher's Exact Test will be used. To compare the means, the Student's *t* test and Analysis of variance (ANOVA) will be used and to analyze the correlation between the continuous variables the test of Pearson correlation will be applied. In the analyzes of the experimental differences in each group the Wilcoxon test will be used. For all analyzes a level of significance of 95% (*P* < .05) will be considered.

## 3 Discussion

Halitosis plays an important role in communication and is related to quality of life and social interaction. Quality of life refers to a person's perception of their position in the system they live in relation to their goals, standard expectations and concerns. It is a comprehensive concept, which is affected by the physical, psychological and social relationship, and relationship with the environment in which it lives. And halitosis is considered one of the problems related to the impact of oral health on quality of life and is enough to affect the individual's perception about his life.^[19]^

Therefore, the need for new treatment alternatives for the patient, especially in the face of the technological advances in dentistry, can work better with the patient's expectation regarding the results.

Because it is a condition that involves mainly anaerobic bacteria, which release the SVC in the exhaled air and are responsible for the bad smell. Its main cause is related to the presence of these microorganisms in the lingual flap. And so, it must work to reduce the number of microorganisms responsible for this problem.

There is still a great deal of divergence in the literature regarding the epidemiological data of this condition due to the lack of standardization in the diagnosis, and the treatment proposed in the literature is broad that consists of the chemical and mechanical reduction of the tongue coating.^[9–13]^

In the initial studies in this area it was possible to observe an immediate result, with the elimination of bad odor by the reduction of CSV concentration specifically of the levels of SH2 when the treatment with photodynamic therapy was used in the dorsum of adolescents with halitosis.^[29–33]^

The present study proposes to conduct a controlled clinical trial to evaluate the effectiveness of the application of aPDT in the tongue coating as a new proposal for treatment and control of halitosis, mainly because it is a fast, non-invasive procedure and does not generate bacterial resistance, as evidenced in the studies cited below and published.

Not generating bacterial resistance can be considered one of the great advantages of aPDT, due to the great problem that we are currently with the development of superbugs being these resistant to the broader antibiotics.

## Author contributions

Conceive and design the study: ACCM, MLLG, SKB, MPAM; will perform the experiment: ACCM, MLLG, LA, ACRTH, AL, ESAS; will analyse the data: AMD, RAMF, KPSF, SKB; will perform the statistical analysis: LJM, AMD; write the paper: ACCM, MLLG, SKB.

**Conceptualization:** Ana Carolina Costa da Mota Ciarcia, Marcela Leticia Leal Gonçalves, Anna Carolina Ratto Tempestine Horliana, Ellen Sayuri Ando Suguimoto, Lysianne Araujo, Andreia Laselva, Marcia Pinto Alves Mayer, Sandra Kalil Bussadori.

**Data curation:** Ana Carolina Costa da Mota Ciarcia, Ellen Sayuri Ando Suguimoto, Lara Jansinsk Motta, Alessandro melo Deana, Raquel Agnelli Mesquita-Ferrari, Sandra Kalil Bussadori.

**Formal analysis:** Ana Carolina Costa da Mota Ciarcia, Lara Jansinsk Motta, Alessandro melo Deana, Raquel Agnelli Mesquita-Ferrari, Kristianne Porta Santos Fernandes, Sandra Kalil Bussadori.

**Funding acquisition:** Sandra Kalil Bussadori.

**Investigation:** Ana Carolina Costa da Mota Ciarcia, Marcela Leticia Leal Gonçalves, Lysianne Araujo, Marcia Pinto Alves Mayer, Sandra Kalil Bussadori.

**Methodology:** Ana Carolina Costa da Mota Ciarcia, Marcela Leticia Leal Gonçalves, Anna Carolina Ratto Tempestine Horliana, Ellen Sayuri Ando Suguimoto, Lysianne Araujo, Andreia Laselva, Marcia Pinto Alves Mayer, Sandra Kalil Bussadori.

**Project administration:** Anna Carolina Ratto Tempestine Horliana, Raquel Agnelli Mesquita-Ferrari, Kristianne Porta Santos Fernandes, Sandra Kalil Bussadori.

**Project administration:** RAMF, KPSF, SKB.

**Resources:** Ana Carolina Costa da Mota Ciarcia, Kristianne Porta Santos Fernandes, Sandra Kalil Bussadori.

**Supervision:** Anna Carolina Ratto Tempestine Horliana, Sandra Kalil Bussadori.

**Validation:** Sandra Kalil Bussadori.

**Visualization:** Sandra Kalil Bussadori.

**Writing – original draft:** Ana Carolina Costa da Mota Ciarcia, Marcela Leticia Leal Gonçalves.

**Writing – review & editing:** Anna Carolina Ratto Tempestine Horliana, Lara Jansinsk Motta, Sandra Kalil Bussadori.

## References

[1] Outhouse TL Al-alawi R Fedorowicz Z Withdrawn: tongue scraping for treating halitosis. Cochrane Database Syst Rev 2016;5:CD005519 Doi: 10.1002/14651858.CD005519.pub3. 10.1002/14651858.CD005519.pub3 PMC10653019 27227886

[2] Calil CM Marcondes FK Influence of anxiety on the production of oral volatile sulfur compounds. Life Science 2006;79:660–4. 10.1016/j.lfs.2006.02.010 16564550

[3] Springfield J Spontaneous fluctuations in the concentrations of oral sulfur-containing gases. J Dental Res 2001;80:1441–4. 10.1177/00220345010800051101 11437216

[4] Tangerman A Winkel EG The portable gas chromatograph OralchromaTM: a method of choice to detect oral and extra-oral halitosis. J Breath Res 2008;2 (1.):10.1088/1752-7155/2/1/017010 21386154

[5] Tolentino EDS Chinellato LEM Tarzia O Saliva and tongue coating pH before and after use of mouthwashes and relationship with parameters of halitosis. J Appl Oral Sci 2011;19:90–4. 21552707 10.1590/S1678-77572011000200002 PMC4243744

[6] Xi Chen Yu Zhang Hai-xia Lu Xi-Ping Feng, Factors associated with halitosis in white-collar employees in Shanghai, China 10.1371/journal.pone.0155592. PMC4871467 27186878

[7] Kara C Effect of Nd: YAG laser irradiation on the treatment of oral malodour associated with chronic periodontitis. Int Dent J 2008;58:151–8. 18630111 10.1111/j.1875-595x.2008.tb00191.x

[8] Kara C Tezel A Orbak R Effect of oral hygiene instruction and scaling on oral malodour in a population of Turkish children with gingival inflammation. Int J Paediatr Dent 2006;16:399–404. 17014537 10.1111/j.1365-263X.2006.00769.x

[9] Bollen CML Beikler T Halitosis: the multidisciplinary approach. Int J Oral Sci 2012;4:55–63. 22722640 10.1038/ijos.2012.39 PMC3412664

[10] Quirynen M Characteristics of 2000 patients who visited a halitosis clinic. J Clin Periodontol 2009;36:970–5. 19811581 10.1111/j.1600-051X.2009.01478.x

[11] Quirynen M Impact of tongue cleansers on microbial load and taste. J Clin Periodontol 2004;31:506–10. 15191584 10.1111/j.0303-6979.2004.00507.x

[12] Raangs G Winkel E Van winkelhoff A In vitro antimicrobial effects of two antihalitosis mouth rinses on oral pathogens and human tongue microbiota. Int J Dent Hyg 2013;11:203–7. 23368885 10.1111/idh.12014

[13] Saad S Greenman J Shaw H Comparative effects of various commercially available mouthrinse formulations on oral malodor. Oral Dis 2011;17:180–6. 20659260 10.1111/j.1601-0825.2010.01714.x

[14] Saad S Hewett K Greenman J Effect of mouth-rinse formulations on oral malodour processes in tongue-derived perfusion biofilm model. J Breath Res 2012;6:016001. 22234955 10.1088/1752-7155/6/1/016001

[15] Pinheiro SL Silva JN Gonçalves RO Manual and rotary instrumentation ability to reduce Enterococcus faecalis associated with photodynamic therapy in deciduous molars. Braz Dent J 2014;25:502–7. 25590196 10.1590/0103-6440201302414

[16] Hope C Wilson M Induction of lethal photosensitization in biofilms using a confocal scanning laser as the excitation source. J Antimicrob Chemother 2006;57:1227–30. 16549510 10.1093/jac/dkl096

[17] Wilson M Lethal photosensitisation of oral bacteria and its potential application in the photodynamic therapy of oral infections. Photochem Photobiol Sci 2004;3:412–8. 15122357 10.1039/b211266c

[18] Wainwright M Photodynamic antimicrobial chemotherapy (PACT). J Antimicrob Chemother 1998;42:13–28. 9700525 10.1093/jac/42.1.13

[19] Lu HX Chen XL Wong M Oral health impact of halitosis in Chinese adults. Int J Dent Hyg 2016;doi: 10.1111/idh.12242. 10.1111/idh.12242 27516401

[20] Pinto EH Longo PL Camargo CCBDE Assessment of the quantity of microorganisms associated with bronchiectasis in saliva, sputum and nasal lavage after periodontal treatment: a study protocol of a randomized controlled trial. BMJ Open 2016;6:e010564 doi:10.1136/bmjopen -2015-010564. 10.1136/bmjopen-2015-010564 PMC4838683 27084279

[21] Rosenberg M Kulkarni G Bosy A Reproducibility and sensitivity of oral malodor measurements with a portable sulfide monitor. J Dent Res 1991;70:1436–40. 1960254 10.1177/00220345910700110801

[22] Rosenberg M Bad breath, diagnosis and treatment. Univ Tor Dent J 1990;3:7–11. 2076696

[23] Kara C Demir T Orbak R Effect of Nd: YAG laser irradiation on the treatment of oral malodour associated with chronic periodontitis. Int Dent J 2008;58:151–8. 18630111 10.1111/j.1875-595x.2008.tb00191.x

[24] Salako NO Philip L Comparison of the use of the Halimeter and the Oral ChromaTM in the assessment of the ability of common cultivable oral anaerobic bacteria to produce malodorous volatile sulfur compounds from cysteine and methionine. Med Princ Pr 2011;20:75–9. 10.1159/000319760 21160219

[25] Vandekerckhove B Van Den Velde S De Smit M Clinical reliability of non-organoleptic oral malodour measurements. J Clin Periodontol 2009;36:964–9. 19843192 10.1111/j.1600-051X.2009.01473.x

[26] Rosenberg M Mcculloch CA Measurement of oral malodor: current methods and future prospects. J Periodontol 1992;63:776–82. 1474479 10.1902/jop.1992.63.9.776

[27] Donaldson AC Riggio MP Rolph HJ Clinical examination of subjects with halitosis. Oral Dis 2007;13:63–70. 17241432 10.1111/j.1601-0825.2006.01248.x

[28] Prates RA Da Silva EG Yamada JR Light parameters influence cell viability in antifungal photodynamic therapy in a fluence and rate fluence-dependent manner. Laser Physics 2009;19:1038–44.

[29] Costa da Mota AC França CM Prates R Effect of photodynamic therapy for the treatment of halitosis in adolescents—a controlled, microbiological, clinical trial. J Biophotonics 2016;9:1337–43. doi: 10.1002/jbio.201600067. 27248838 10.1002/jbio.201600067

[30] Gonçalves M Bussadori S Fragoso Y Effect of photodynamic therapy in the reduction of halitosis in patients with multiple sclerosis—clinical trial. J Breath Res 2017;doi: 10.1088/1752-7163/aa8209. 10.1088/1752-7163/aa8209 28742057

[31] Lopes RG Da Mota AC Soares C Immediate results of photodynamic therapy for the treatment of halitosis in adolescents: a randomized, controlled, clinical trial. Lasers Med Sci 2016;31:41–7. Doi: 10.1007/s10103-015-1822-6. 26510574 10.1007/s10103-015-1822-6

[32] Lopes RG De Santi ME Franco BE Photodynamic therapy as a novel treatment for halitosis in adolescents: a case series study. J Lasers Med Sci 2014;5:146–52. 25653814 PMC4281993

[33] Lopes RG De Godoy CH Deana AM Photodynamic therapy as a novel treatment for halitosis in adolescents: study protocol for a randomized controlled trial. Trials 2014;15:443 Doi: 10.1186/1745-6215-15-443. 25394474 10.1186/1745-6215-15-443 PMC4236439

